# Recovery of Bioactive Compounds from Hazelnuts and Walnuts Shells: Quantitative–Qualitative Analysis and Chromatographic Purification

**DOI:** 10.3390/biom10101363

**Published:** 2020-09-24

**Authors:** René Herrera, Jarl Hemming, Annika Smeds, Oihana Gordobil, Stefan Willför, Jalel Labidi

**Affiliations:** 1 Chemical and Environmental Engineering Department, University of the Basque Country (UPV/EHU), Plaza Europa 1, 20018 San Sebastián, Spain; jalel.labidi@ehu.es; 2 InnoRenew CoE, Livade 6, 6310 Izola, Slovenia; oihana.gordobil@innorenew.eu; 3 Chemistry and Chemical Engineering Department, Åbo Akademi University, Process Chemistry Centre, Porthansgatan 3, FI-20500 Åbo, Finland; jarl.hemming@abo.fi (J.H.); annika.smeds@abo.fi (A.S.); swillfor@abo.fi (S.W.)

**Keywords:** nutshells, biowaste valorization, accelerated extraction, chromatographic analysis, fractionation, phenolic compounds, antioxidant capacity

## Abstract

Hazelnut (HS) and walnut (WS) shells, an abundant by-product of the processing industries of these edible nuts, are traditionally considered as a low-value waste. However, they are a source of valuable compounds with an interesting chemical profile for the chemical and pharmaceutical sectors. In this study, the lipophilic and hydrophilic extracts present in HS and WS were quantified and identified, then the polar fractions were chromatographically separated, and their antioxidant capacity was studied. The experimental work includes the isolation of crude lipophilic and hydrophilic extracts by an accelerated extraction process, chromatographic analysis (gas chromatography-flame ionization (GC-FID), GC-mass spectroscopy (GC-MS), high-performance size-exclusion chromatography (HPSEC), thin-layer chromatography (TLC)), and quantification of the components. In addition, a thorough compositional characterization of the subgroups obtained by flash chromatography and their antioxidant capacity was carried out. The gravimetric concentrations showed different lipophilic/hydrophilic ratios (0.70 for HS and 0.23 for WS), indicating a higher proportion of polar compounds in WS than in HS. Moreover, the lipophilic extracts were principally composed of short-chain fatty acids (stearic, palmitic, and oleic acid), triglycerides, and sterols. The polar fractions were screened by thin-layer chromatography and then separated by flash chromatography, obtaining fractions free of fatty acids and sugar derivatives (97:3 in HS and 95:5 in WS), and mixtures richer in phenolic compounds and flavonoids such as guaiacyl derivatives, quercetin, pinobanksin, and catechin. The most polar fractions presented a higher antioxidant capacity than that of the crude extracts.

## 1. Introduction

Nowadays, there is an increasing demand for petrochemical-free and safe products. The potential of the agroforestry residues in terms of the diversity of natural compounds that can be obtained is very broad and involves markets such as the pharmaceutical, cosmetic, food, and additives, among others [1,2]. Therefore, the agroforestry sector has a great opportunity to increase the overall mobilization rate due to the higher added value of the output products. In this context, the obtaining of a range of chemical compounds from sustainable sources but focused on the valorization of industrially generated by-products represents an interesting research target with environmental and economic benefits, promoting the development of the circular bioeconomy [3].

Hazelnuts (*Corylus avellana* L. Betulaceae) and walnuts (*Juglans regia* L. Juglandaceae) are important commercial crops worldwide, which generate a large amount of shells as by-products from the processing industry [4,5]. On the one hand, hazelnuts are well-known nuts with an average annual production of about 55,8500 tons (with shell), out of which 75% is produced in Turkey, followed by 15% in Italy [6]. On the other hand, walnut trees are globally cultivated for their edible kernels, which are enclosed in a hard shell and according to FAO (2018), the annual production is approximately 3.66 million tons (with shell), with China being the principal producer (around 30%), followed by Iran, USA, and Turkey [6]. 

The shells represent between 50% and 70% of the nut weight and are chemically composed of hemicelluloses (22–30%(*w*/*w*)), cellulose (25–28%(*w*/*w*)), lignin (40–50%(*w*/*w*)), and others (up to 7%) [7]. However, despite their high potential as a source of chemicals, these agricultural residues are traditionally used as a solid fuel for heating. More recently, the use of nutshells has been investigated to produce sugars and methanol, as well as being a source of natural antioxidant compounds [8,9,10,11].

Moreover, at the present time, there is a growing demand to replace synthetic products from non-renewable sources with products from biomass. As an example, since antioxidant compounds are present in most of the products consumed today, an increasing demand for natural antioxidants as an alternative to artificial ones has recently been observed [12,13]. Antioxidant activity is associated with the presence of bioactive components like phenolic compounds, which have positive effects on human health by reducing oxidative stress and inhibiting macromolecular oxidation [14].

Additionally, phenolic compounds have exhibited biological and physiological properties, including antiallergenic, antiatherogenic, anti-inflammatory, antimicrobial, and antithrombotic properties [15]. Several industrial sectors demand active chemical compounds as additives of their final products. The inherent beneficial properties of natural products are relevant for the cosmetic and health care products, pharmaceutical, and food industries, increasing the research interest in the development of effective extraction techniques to improve the product yields, while reducing time and solvent consumption [16].

Traditional direct extraction methods with hot or room temperature solvents are the most commonly used to obtain natural extracts from plants. However, the use of organic solvents may lead to an environmental problem due to the large amounts of solvents required. Moreover, novel extraction methods including ultrasound-assisted extraction, microwave-assisted extraction, supercritical fluid extraction, autohydrolysis, ohmic heating, and pressurized liquid extraction have been investigated as relatively efficient and cost-effective alternatives to the traditional methods [16,17]. 

Among these methods, accelerated solvent extraction (ASE) is an excellent technique which slightly increases the extraction yields in comparison to the techniques mentioned before [18,19]. The ASE is performed under an inert atmosphere at an elevated temperature and pressure using small solvent volumes, thus minimizing the thermal degradation and isomerization of the extracts. This technique also allows sequential extractions in short times and is automated, which is a great advantage when many samples are extracted with different solvents. 

Although the shells of these nuts could be a promising feedstock for bioactive compounds, there is a lack of knowledge about the chemical composition of their lipophilic and hydrophilic extracts. Moreover, a better insight into their possible applications will be provided by knowing the potential bioactivity of their component groups, thus giving an added value over the current use of these agroforestry residues. The objectives of this study were to extract, quantify, and identify the lipophilic and hydrophilic compounds present in hazelnuts and walnuts shells, and then to fractionate the polar compounds by flash chromatography in order to study possible bioactivities present in the obtained groups of compounds.

## 2. Materials and Methods

### 2.1. Materials and Preparation

**Figure 1:**
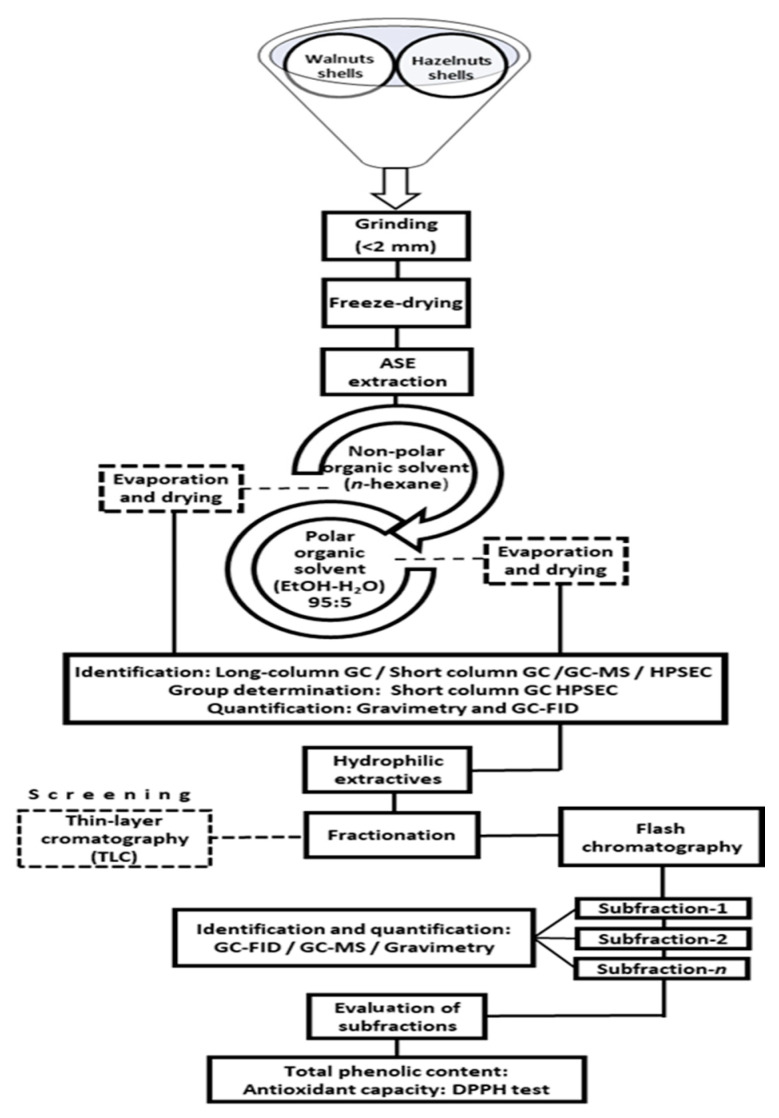
Protocol for extraction, fractionation, and evaluation of nutshells.

The hazelnuts (*Corylus avellana*) and walnuts (*Juglans regia*) shells used in this study were provided by local nut producers from the Basque Country (Northern Spain). Selected shells were first ground to pass through a 10-mesh (2-mm) sieve, using a Retsch Hammer mill, and stored at a low temperature in airtight bags. Afterwards, shells were freeze-dried for a few days and then milled to a mean particle size of 1 mm (18-mesh, sieve, Retsch SM-100 mill) to finally freeze-dry again (Figure 1). The freeze-drying process was applied twice to remove possible water uptake during the milling processes and thus to avoid any degradation of the material during the storage. The grinded samples were stored in airtight bags at −18 °C for further analysis.

### 2.2. Crude Extracts from Shells

The isolation of extracts from shell samples (approx. 4 g per sample) were carried out in a rapid sequential ASE apparatus (Accelerated Solvent Extractor, Dionex Corp., Sunnyvale, FL, USA). The ASE was programmed to pour first hexane and then acetone-water (95:5) as eluents. The lipophilic extracts were collected with hexane (solvent temperature 90 °C, pressure 13.8 MPa, 2 × 5 min static cycles) and subsequently, the hydrophilic extracts were collected with the acetone-water solvent (solvent temperature 100 °C, pressure 13.8 MPa, 2 × 5 min static cycles) [20,21]. From each sample, 50 of lipophilic solution and 50 mL of hydrophilic solution were obtained. An aliquot of 10 mL of each solution was taken to calculate the gravimetric concentration by evaporating the samples until dryness using N_2_ and then drying in a vacuum desiccator at 40 °C (dry weight). The concentration was calculated as mg of extracts per gram of dry raw material (shells).

### 2.3. Analysis of Raw Extracts

The lipophilic and hydrophilic fractions were prepared for their derivatization (by silylation reaction) to analyze their composition by a gas chromatography-flame ionization detection (GC-FID) technique. First running a classical GC column (25 m × 0.20 mm column coated with crosslinked methyl polysiloxane, 0.11 µm film thickness) and then a short column (6 m × 0.53 mm column coated with crosslinked methyl polysiloxane, 0.15 µm film thickness).

The peak areas in the chromatograms were identified as silylated derivatives and transformed into a concentration of each component identified by GC-mass spectroscopy (HP 6890-5973 GC-MSD equipment) using the in-house Spectral Library and the commercial Wiley 10th/NIST 2012 spectral library. The compounds were organized into component groups and the practical limit of quantification of the individual compounds was about 0.01 mg/g [22].

Additionally, high-performance size-exclusion chromatography (HPSEC-ELSD system) was performed for hydrophilic extracts to observe the molecular mass distribution of the principal groups present in the polar fraction. The HPSEC equipment (Shimadzu Corporation, Kyoto, Japan) consisted of a LC-10ATVP pump, a DGU-14A on-line degasser, aSCL-10AVP system controller, a SIL-20 AHT autosampler, aCTO-10ACvp column oven, and a SEDEX85 LF low-temperature evaporative light scattering detector ELSD (SEDERE S.A., Alfortville Cedex, Alfortville, France). The columns were two Jordi Gel DVB 500A (300 × 7.8 mm) in series (Columnex LLC, New York, NY, USA), equipped with a guard column (50 × 7.8 mm). The eluent was THF/AcOH 99:1(*v*/*v*) with a flow rate of 0.8 mL min^−1^ and the analysis time was 28 min. The injection volume was 100 μL and the concentration of samples was adjusted to 1 mg∙mL^−1^ of the extract solution. 

### 2.4. Fractionation and Characterization of the Hydrophilic Extracts

As a preliminary step, thin-layer chromatography (TLC) was applied to determine the adequate eluent and ratios to fractionate the extracts [23]. The most effective mobile phase observed with TLC was dichloromethane-ethanol (DCM-EtOH); thus, a stepwise gradient from 97:3 to 90:10 (*v*/*v*) was applied for the subsequent fractionation process. 

The fractionation was carried out using a normal-phase silica Biotage flash 40i chromatography column (Biotage UK Ltd., Hertford, England) with a flow rate from 30 to 50 mL and a load capacity of 2000 to 3500 mg. Firstly, the raw hydrophilic extract was evaporated using a rotavapor and then was homogenously mixed with the silica in a flash cartridge (Biotage Si 40M). An initial cleaning step with hexane was applied and then the separation was performed with different gradients of DCM-EtOH eluent at a flow rate of 30 mL/min. Fractions of 50 mL were collected and examined again by TLC. Aliquots of the most interesting fractions were evaporated and silylated for the GC-FID, GC-MS characterization. Finally, the fractions were stored for further analysis.

### 2.5. Preliminary Tests of TPC and Antioxidant Activity of the Hydrophilic Fractions

The total phenolic content (TPC) of the hydrophilic fractions was evaluated by the Folin–Ciocalteu spectrophotometric method using gallic acid as a reference compound and dimethyl sulfoxide (DMSO) as a solvent [24]. Extracts were dissolved in DMSO (1 mg/mL). Then, 0.5 mL of diluted extract samples were mixed with 2.5 mL of Folin’s reagent (Sigma–Aldrich, Madrid, Spain) (diluted with distilled water 1:10) and 2 mL of 7.5% (*w*/*v*) sodium bicarbonate solution. The absorbance was determined spectrophotometrically at 725 nm using a microplate reader (Epoch 2, BioTek, Winooski, VT, USA) after standing for 60 min at room temperature. The results were expressed as milligram gallic acid equivalent (GAE) per gram of extract. 

The DPPH radical scavenging activity assay was prepared by dissolving 3.5 mg of DPPH radical in 100 mL of ethanol as DPPH radical solution and dissolving the extracts in methanol. Then, 1 mL of the prepared DPPH solution was added to 50 µL of the diluted extract sample [25]. The absorbance was measured at 517 nm with a microplate reader (Epoch 2, BioTek, Winooski, VT, USA) after 30 min of incubation at room temperature. The commercial antioxidant butylated hydroxytoluene (BHT) was used as reference standards. All measurements were performed in triplicate. The percentage of remaining DPPH was plotted against the sample/standard concentration to obtain IC50 value, which represents the concentration of the extract or standard antioxidant (mg/mL) required to scavenge 50% of the DPPH in the reaction mixture. The antiradical power (ARP, ARP = 1/IC50) was also calculated.

### 2.6. Statistical Analysis

The gravimetric values of the raw extracts and fractions, TPC and DPPH radical scavenging activity were performed by triplicate to perform an analysis of variance (ANOVA), where the differences with *p* < 0.05 were considered significant, and an additional Bonferroni significant difference (BSD) was applied after rejecting the null hypothesis. The software used for the statistical and graphing analysis was OriginPro 2015 (V. 9.2).

## 3. Results and Discussion

### 3.1. Extracts Yields and Chemical Composition

The ASE technique allows for the recovery of the polar and lipophilic fraction separately, facilitating its quantification and chromatographic analysis (Table 1). The gravimetric concentration of the extracts from the hazelnuts and walnuts shells was uneven, obtaining the maximum extraction yield from walnuts shells (WS), which was approximately 4.5 mg/g greater than in hazelnuts shells (HS). In addition, the hydrophilic compounds appeared in a higher proportion in both nutshells (59% HS and 81% WS).

Subsequently, the obtained fractions were analyzed by gas chromatography (GC), finding a reduced concentration compared to the gravimetric values, as well as changes in the L/H ratio, which was increased in HS and slightly reduced in WS. This gap between gravimetric and GC quantification could be attributed to the complex mixtures of compounds with low and high molar mass that are present in the extracts and to polymerization reactions during the extraction process [26]. Therefore, GC facilitates the separation of components with a low molar mass, but high molar mass components are not readable [27]. Apparently, below C60 the separation is acceptable on short thin-film columns; thus, the lipophilic fraction up to triacylglycerols and the hydrophilic fraction up to four lignan units could be successfully analyzed by GC [23,28]. For high-molar mass components, the HPLC analysis is more convenient; however, the goal of this study was to classify, separate, and identify non-volatile extracts, omitting polysaccharides and other high molar mass components.

**Figure 2:**
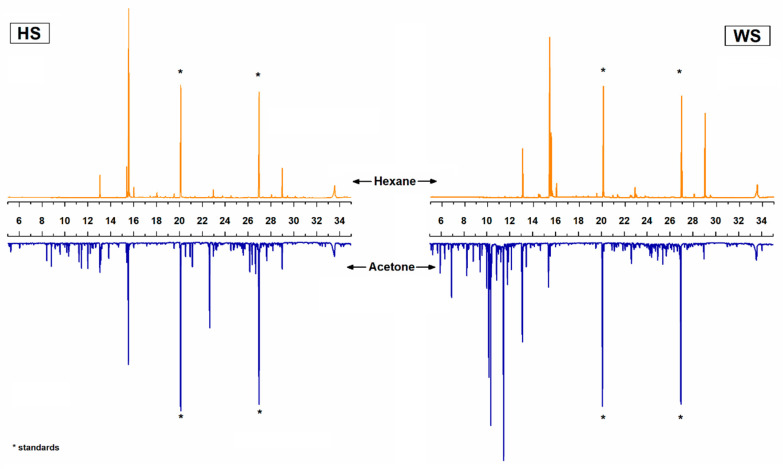
Classical GC column chromatograms of the lipophilic and hydrophilic fractions of hazelnut shell (HS) and walnut shell (WS) extracts.

In Table 2, the chemical constituents from the lipophilic and polar fractions determined by GC-mass spectroscopy (Figure 2) and grouped according to their chemical structure are summarized. Regarding the lipophilic fractions extracted with hexane, they were mainly composed by short-chain fatty acids (up to 20-C atoms) and glycerides (mainly triglycerides), followed by long-chain fatty acids (up to 28-C atoms) and steroids (sterols and steryl esters), and finally, minor amounts of terpenes and sugar-derived compounds.

Moreover, the major lipophilic compounds identified in HS from the largest to smallest amount were: Oleic acid (2.16 mg/g), linoleic acid (0.30 mg/g), sitosterol (0.26 mg/g), palmitic acid (0.19 mg/g), and stearic acid (0.10 mg/g). On the other hand, the main compounds found in WS from the largest to smallest amount were: Linoleic acid (0.82 mg/g), sitosterol (0.41 mg/g), oleic acid (0.30 mg/g), palmitic acid (0.21 mg/g), and stearic acid (0.06 mg/g). In general, the principal groups found in the lipophilic fraction of the walnut and hazelnut shells appear in most of the shells of the edible nuts, which are mostly identified unsaturated (stearic, palmitic acid) and saturated (oleic acids) fatty acids, large concentrations of sterol (dominated by sitosterol), glycerides, and terpenoids, and they can be recovered for their utilization as tall oil and sterol-based precursors [28,29]. 

The second fraction, a hydrophilic fraction, was consecutively eluted with acetone-water (95:5), obtaining higher yields (Table 1), as well as a larger list of chemical compounds, as shown in Table 2, Table 3 and Table 4. The concentration of polar components was significantly higher in WS than in HS, following the trend of the gravimetric results (Table 2), and was composed mainly by phenolic compounds (phenols, phenolic acids, lignans, flavonoids, and stilbenes), sugar derivatives, resin acids, glycerides, and remaining fractions of fatty acids (mainly oleic acids).

Some differences were found between the hydrophilic fractions of the studied materials, and in the case of HS, resin acids were identified as the major components (mainly modified dehydroabietic acids), followed by sugar derivatives, glycerides, and phenolic compounds. On the other hand, the components of WS were preceded by sugar derivatives (mainly monosaccharides), phenolic compounds (hydroxybenzoic and phenolic acids), and modified dehydroabietic acids. These general results show a large number of compounds in the hydrophilic fraction with a wide range of molecular weights, making it interesting to have a more detailed exploratory analysis and an additional separation process of the potential bioactive compounds.

**Figure 3:**
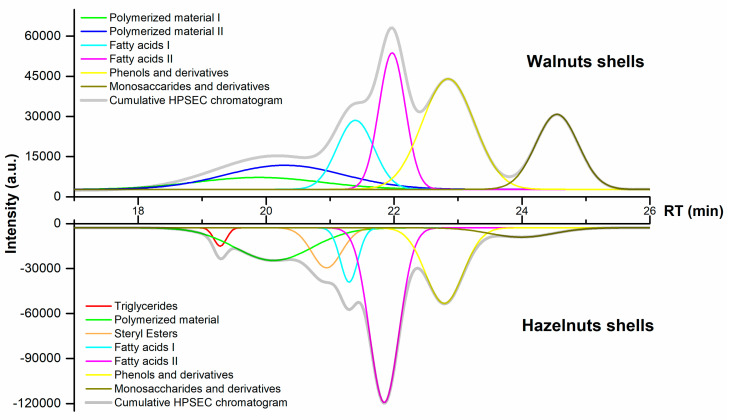
High-performance size-exclusion chromatography (HPSEC) chromatograms and deconvoluted signals from the polar extracts.

Therefore, the molecular weight distribution of the hydrophilic fractions was screened by HPSEC in order to observe their polydispersity. Nevertheless, the raw chromatograms hardly displayed the group distribution, due to overlapping signals, intensity, and retention times. For this reason, the raw chromatograms were deconvoluted by removing part of the overlapping signals and improving the signal-to-noise ratios. The cumulative HPSEC and its individual signals are observed in Figure 3, and the group assignment was set considering the database at ÅAU along with references [30,31].

The deconvoluted signals at the earlies retention times (up to 21 min) show unknown peaks that could be attributed to oligomers or polymerized material. Phenols and derivatives and monosaccharide derivatives appear at the latter retention time within the same chromatographic range. The peak areas are relative because some compounds are not easily soluble in the elution solvent (like sugars), and some phenolics are too volatile to be detected by the ELSD detector.

The group assignment was comparable in both shells, including groups from long- to short-chain fatty acids, phenols derivatives (lignans, resin acids, and flavonoids) and monosaccharides. However, two peaks were observed in the HS at early retention times (between 19–19.5 and 20.5–21.5), which could be attributed to triglycerides and steryl esters [30,32]. The resolution of these chromatograms was not enough to clearly observe the peaks from individual components, but this overview was useful to know the distribution of some target compounds that were subsequently separated by means of flash chromatography.

### 3.2. Isolation of Component Groups from Hydrophilic Fractions

The solvent selection and their adequate ratios were pre-selected by using thin-layer chromatography (TLC). This technique was used due to its simplicity in screening the performance of several eluents on different samples [23,33]. The results showed that the mixture of DCM-EtOH 93:7 (*v*/*v*) appeared as the most suitable eluent to separate the polar groups. Thereby, the experimental isolation process was planned considering the selected eluent at different ratios, starting the separation from lower (97:3) to higher polarities (90:10), on normal-phase silica columns.

**Figure 4:**
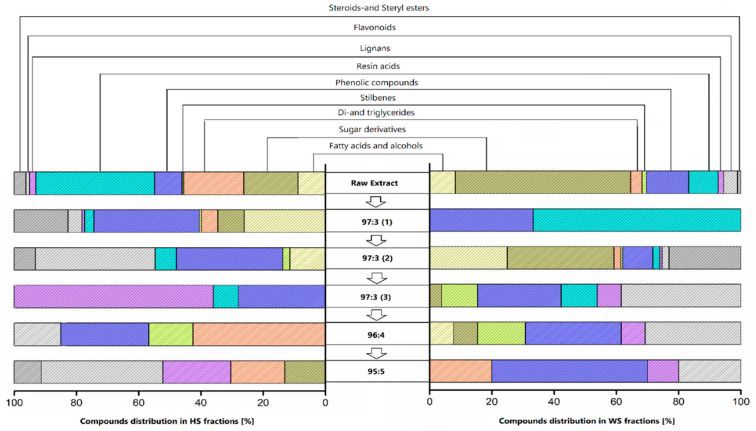
Groups distribution of crude extract and their fractions after separation by flash chromatography.

From the different elution steps, the subfractions were collected, and particularly the solvent ratio 97:3 was collected in 3 different batches due to the larger number of compounds. On the one hand, HS presented the following order from the largest to the lower concentration: 97:3(1) (82%)→97:3(2) (6%)→97:3(3) and 96:4 (4.2% each one)→ 95:5 (3.9%). On the other hand, WS showed the following order: 97:3(2) (85%)→ 97:3(3) (8%)→ 96:4 (4%)→ 95:5 (1%)→ 97:3(1) (0.1%). The separated fractions were characterized by GC and GC-MS, and the description of the component groups found in each elution step is presented in Table 3 (HS) and Table 4 (WS). The group distribution on each fraction is synthetized in Figure 4.

The components’ distribution on each HS fraction is observed in Table 3. The most concentrated fraction recovered from HS was 97:3(1), and mainly contained a wide range of phenolic compounds (phenols, phenolic acids, flavonoids), linoleic acid, sitosterol, and a smaller amount of sugar derivatives and glycerides. The following fraction, 97:3(2), also presented a high concentration and consisted of mixtures of phenolic compounds (remarkable amount of quercetin), resin acids, sitosterol, and a smaller amount of fatty acids. Consecutive fraction, 97:3(3), contained guaiacyl derivatives, lignans, and resin acids, and phenolic compounds like guaiacyl derivatives in the case of 96:4, while 95:5 was mainly composed of lignans (todolactol) and flavonoids (pinobasquin). Previous research studies also reported the presence of oleic and linoleic acids, phenolic compounds like gallic acid, vanillin, vanillic acid, guaiacyl derivatives, and flavonoids such as catechin and quercetin. The results were compared to other ligneous co-products, like almond and pine shells, and chestnut peel. These studies also reported the presence of phenolic compounds, flavonoids, and sterols (such as gallic acid, catechin, and vanillin) [34,35,36,37]. However, the fractionation of the pure extracts and their GC quantifications [mg/g] were not performed by other authors, which did not allow a direct comparison. 

The composition of the first WS fraction 97:3(1) appeared at a very low concentration (Table 4), with moieties of resin acids and phenols. However, the following fraction, 97:3(2), presented a complex mixture of compounds with high concentrations of sugar derivatives (though much lower than raw extracts), fatty acids, and sitosterol, and a reduced concentration of phenolic compounds. In the following fractions the principal compounds were reduced to phenolic compounds (phenols, phenolic acids, stilbenes, and flavonoids), and it is worth mentioning gallocatechin, pinocembrin, todolactol, and 1-syringylglycerol, as compounds that were present in these fractions. Recently, the presence of phenolics and flavonoids in polar extract from walnut shells isolated by solvent extraction with ethanol:water has been reported [36]. However, no prior analysis reporting data about the identification and quantification of the chemical constituents present in this type of residue has been reported.

### 3.3. TPC and Antioxidant Activity of Isolated Fractions from Crude Hydrophilic Extracts

The results of total phenolic content (TPC) and antioxidant activity (IC50) of the polar extracts and isolated fractions are observed in Table 5. The TPC of HS crude polar extract was significantly higher (41.75) than the one observed for WS hydrophilic extract (13.14 mg GAE/g extract). The reported data from this type of lignocellulosic extracts vary greatly since the chemical composition is highly influenced by the isolation method and solvent type employed [5,34,36,38]. However, similar results were found for hydrophilic extracts from hazelnut shells isolated by methanol (56.6), ethanol (59.6), and acetone (72.2 mg GAE/g) [5]. Moreover, for extracts derived from walnuts shells lower TPC values were found ranging from 13.4 to 30.1 mg GAE/g extract [39], which is in agreement with the results of the present work. Regarding the polar fractions, as can be observed, TPC values were increased, especially for the most polar fractions. The main characteristics of these fractions are the total removal of fatty acids, sitosterol, and other compounds such as sugar derivatives, which could cause a negative effect on the determination of TPC.

Finally, the antioxidant activity of the extracts and their fractions was evaluated by the DPPH indirect method, which is commonly used as a free radical to evaluate the antioxidant activity of natural extracts due to its stability, simplicity, and reproducibility [40]. The results demonstrated a significant improvement in the antioxidant activity after the fractionation process. The high antioxidant capacity showed by 97:3 (3), 96:4, and 95:5 fractions of HS was due to their homogeneous chemical composition practically reduced to one or two group components. As it is reported in Table 3, the main components of 97:3 (3) were todolactol and hydroxymatairesinol (HMR), which are considered powerful antioxidants [40,41]. Moreover, the 96:4 fraction was mainly composed of phenolic compounds, while the composition of 95:5 was based on todolactol and flavonoids such as pinobasquin and catechin. Furthermore, the first two fractions of WS (97.3 (1) and 97:3 (2)) did not show antioxidant activity. However, a clear improvement was found for other fractions, which were basically composed of phenolic compounds and flavonoids. In general, it is possible to affirm that fractions with a higher amount of fatty acids and sugar derivatives diminish the antioxidant effect of samples.

## 4. Conclusions

In this work, hazelnuts and walnuts shells, which are industrially generated as waste, were used as a source of natural compounds. Lipophilic and hydrophilic extracts were extracted, identified, and quantified. Several differences were found in the yields and composition of isolated raw extracts, especially in the hydrophilic fractions. The lipophilic fractions were mainly composed of fatty acids and alcohols, glycerides, sterols and steryl esters, and moieties of resin acids and terpenoids. However, the hydrophilic fractions were more complex mixtures containing mainly sugar derivatives, phenolic compounds, resin acids, diglycerides, flavonoids, and remaining fractions of oleic acids. The major chemical constituents found in the hydrophilic fraction of WS from highest to lowest were sugar derivatives, phenolic compounds, resin acids, fatty acids, and flavonoids, while in the case of HS the principal components were resin acids, triglycerides, phenolic compounds, sugar derivatives, and fatty acids. The fractionation of hydrophilic raw extracts using silica flash chromatography provided more homogeneous fractions, reducing some fractions to practically only the presence of phenolic compounds (phenols, phenolic acids, stilbenes) and flavonoids. Moreover, fractions, especially the most polar ones, were totally free of fatty acids, sugar derivatives, and triglycerides. This was clearly reflected in the TPC analysis and antioxidant test, where the most polar fractions showed great improvement against their crude extract. Finally, hazelnuts and walnuts shells could be utilized as a raw material for the obtention of high-value molecules with broad potential applications. Vanillin, gallic acid, todolactol, quercetin, catechin, gallocatechin, tyrosol, and resorcinol were the most significant molecules found in this study. These molecules are of great importance as health-promoting ingredients in the food and pharmaceutical industries. Moreover, they could be employed in cosmetic formulations and skin photoprotection applications.

## Figures and Tables

**Table 1:** Gravimetric analysis of lipophilic and polar extracts.

Sample	Extracted Groups	Gravimetric Quantification (mg/g) *	GC Quantification (mg/g) *
Hazelnuts shells	Lipophilic (L)	9.64 ± 0.43	7.01
	Hydrophilic (H)	13.80 ± 0.74	7.88
	Total	23.44	14.89
	L/H ratio	0.70	0.89
Walnuts shells	Lipophilic (L)	5.24 ± 0.18	3.84
	Hydrophilic (H)	22.68 ± 1.37	17.80
	Total	27.92	21.64
	L/H ratio	0.23	0.21

* mg of extracts per g of shells; standard deviation (*n* = 3).

**Table 2:** Groups of components present in the fractions (lipophilic and polar) of HS and WS determined by gas chromatography (GC) and GC-mass spectroscopy (GC-MS).

Component Groups		Hazelnut Shell (mg/g)			Walnut Shell (mg/g)		
		Lipophilic	Hydrophilic	Total	Lipophilic	Hydrophilic	Total
Fatty acids and alcohols	Unsaturated	2.58	0.60	3.18	1.21	0.66	1.87
	Saturated	0.45	0.03	0.48	0.35	0.29	0.64
	Fatty alcohols	0.01	-	0.01	0.02	0.40	0.42
	Subtotal	3.04	0.63	3.67	1.58	1.35	2.93
Terpenes	Terpenoids	0.03	0.02	0.05	0.01	0.06	0.07
Resin acids	Resin acids	0.03	0.16	0.19	0.01	0.12	0.13
	Modified r.a.	-	2.60	2.60	-	1.42	1.42
	Subtotal	0.06	2.78	2.84	0.02	1.60	1.62
	Diglycerides	0.26	1.05	1.31	0.23	0.45	0.68
Glycerides	Triglycerides	3.13	0.33	3.46	1.40	0.13	1.53
	Subtotal	3.39	1.38	4.77	1.63	0.58	2.21
Steroids	Sterols	0.34	0.10	0.44	0.45	0.17	0.62
	Steryl esters	0.10	0.17	0.27	0.10	-	0.10
	Subtotal	0.44	0.27	0.71	0.55	0.17	0.72
Phenolic compounds	Phenols, phenolic acids and lignin units	-	0.64	0.64	-	2.22	2.22
	Lignans	-	0.14	0.14	-	0.30	0.30
	Stilbenes					0.01	0.02
	Flavonoids	-	0.09	0.09	-	0.69	0.69
	Unknown moieties	-	0.03	0.03	-	0.23	0.23
	Subtotal	-	0.90	0.90	-	3.49	3.49
Other compounds	Sugars and derivatives		0.66	0.66	0.01	9.21	9.22
	Others	0.03	0.62	0.65	0.02	1.27	1.29
	Subtotal	0.03	1.28	1.31	0.03	10.48	10.51
Non-identified		0.05	0.64	0.69	0.03	0.13	0.16
	TOTAL	7.01	7.88	14.89	3.84	17.80	21.64

**Table 3:** Composition of crude HS extract and dichloromethane-ethanol (DCM-EtOH) fractions.

Compounds in Hazelnut Shell		Extract *	Fractions DCM-EtOH (mg/g)				
Fatty Acids and Alcohols			97–3 (1) *	97–3 (2) *	97–3 (3) *	96–4 *	95–5 *
Fatty acids	Acid 9:0	0.01 ^a^	-	-	-	-	-
	Acid 18:0	0.02 ^a^	0.21 ± 0.02	-	-	-	-
	Acid 18:1	0.45 ± 0.03	-	0.04 ^a^	-	-	-
	Acid 18:2	0.09 ± 0.01	1.03 ± 0.14	0.01^a^	-	-	-
**Terpenes and Terpenoids**							
	*p*-menthane-1,8-diol	0.02 ^a^	-	-	-	-	-
Di-and triglycerides							
	Diglycerides	1.05 ± 0.08	0.25 ± 0.01	-	-	0.02 ^a^	0.04 ^a^
	Triglycerides	0.33 ± 0.03	-	-	-	0.01^a^	-
**Resin Acids**							
Resin acids	Dehydroabietic acid	0.02 ^a^	-	-	-	-	-
	Pimaric acid	-	0.03 ^a^	-	-	-	-
	Unidentified RT = 21:59–27:75	0.14 ^a^	0.03^a^	0.03 ^a^	0.01 ^a^	-	-
Modified resin acids	7-OH-dehydroabietic (DHA) acid	0.47 ± 0.04	-	-	-	-	-
	X-OH-7oxoDHA acid	-	0.03 ^a^	-	0.01 ^a^	-	-
	Dihydroxy-DHA acid	2.12 ± 0.21	-	-	-	-	-
	9-hydroxystearic acid	0.01 ^a^	0.05 ^a^	-	-	-	-
**Steroids**							
	Sitosterol	0.10 ^a^	0.81 ± 0.06	0.02 ^a^	-	-	-
	Steryl esters	0.17 ± 0.01	0.01^a^	0.01 ^a^	-	-	0.02 ^a^
**Phenolic Compounds**							
Phenols	Vanillin	0.11 ^a^	0.38 ± 0.03	-	-	-	-
	3,4-dihydroxybenzaldehyde	0.02 ^a^	-	-	-	-	-
	3-methoxy-4-OH-cinnamaldehyde	0.03 ^a^	0.19 ^a^	-	-	-	-
	Gallic acid	0.01 ^a^	0.78 ± 0.07	0.02 ^a^	-	-	-
	Protocatechuic acid	0.04 ^a^	-	-	-	-	-
	Dihydroconiferyl alcohol	0.01 ^a^	0.02^a^	-	-	-	-
	Vanillic acid	0.07 ^a^	-	-	0.03 ^a^	0.03 ^a^	-
Phenolic acid	4-hydroxybenzoic acid	0.01 ^a^	-	-	-	-	-
	1-guaiacyl-3-OH-1-propanone	0.04 ^a^	0.01 ^a^	0.07 ^a^	0.04 ^a^	-	-
	1-guaiacyl-2-OH-1-ethanone	0.01 ^a^	0.02 ^a^	-	-	-	-
	1-syringylglycerol	-	-	-	-	0.02 ^a^	-
	1-guaiacyl -2,3-diOH-1-propanone	0.05 ^a^	0.01 ^a^	-	-	0.07 ^a^	-
	1-guaiacylglycerol	0.07 ^a^	-	-	-	-	-
	1,3-(bis-guaiacyl)-1,2-propandiol	0.08 ^a^	0.01 ^a^	-	-	0.07 ^a^	-
	*P*-hydroxyphenyl glycerol	-	-	-	-	0.01 ^a^	-
	Coniferyl alcohol	0.07 ^a^	0.18 ± 0.02	0.01 ^a^	-	-	-
	Monomethyl pinosylvin	0.01 ^a^	0.01 ^a^	-	-	-	-
	4,4′-diOH-3,3′-dimethoxystilbene	0.01 ^a^	-	-	-	-	-
Stilbenes and diarylheptanoids	pinosylvin	0.01 ^a^	-	-	-	-	-
	X-c4h4(oh)3; c5h6(oh)3	0.03 ^a^	0.03 ^a^	0.01 ^a^	-	0.01 ^a^	-
Lignans	Olivil	0.01 ^a^	0.04 ^a^	-	-	-	-
	glucopyranoside2[-4(OH)phe]	0.01 ^a^	-	-	-	-	-
	HMR	-	-	-	0.08 ^a^	-	-
	Todolactol	0.12 ± 0.02	-	-	0.08 ^a^	-	0.05 ^a^
Flavonoids	Pinobanksin	0.05 ^a^	-	-	-	-	0.05 ^a^
	(+)-Catechin	0.01 ^a^	0.08 ^a^	-	-	0.01 ^a^	0.02 ^a^
	Quercetin	0.02 ^a^	0.12 ± 0.01	0.10 ± 0.01	-	-	-
	Gallocatechin (1)	0.01 ^a^	-	-	-	-	-
	Unidentified RT = 27.09–27:80	-	0.01 ^a^	-	-	-	0.02 ^a^
**Other Compounds**							
Sugar derivatives	Monosaccharides	0.60 ± 0.08	0.40 ± 0.05	-	-	-	0.03 ^a^
	Sugar acids	0.02 ^a^	-	-	-	-	-
	Sugar alcohol	0.02 ^a^	-	-	-	-	-
Others	Carboxyl acid	0.58 ± 0.06	-	-	-	-	-
	Alpha-lapachone	0.02 ^a^	-	-	-	-	-
	Dehydro-alpha-lapachone	0.01 ^a^	-	-	-	-	-
	Maltol	0.01 ^a^	-	-	-	-	-
	Non-identified	0.64 ± 0.11	0.15 ± 0.04	-	-	-	-

Standard deviation (*n* = 3); ^a^ s.d. < 0.01. * mg of extracts per g of shells.

**Table 4:** Composition of crude WS extract and DCM-EtOH fractions.

Compounds in Walnut Shell		Extract *	Fractions DCM-EtOH				
Fatty Acids and Alcohols			97–3 (1) *	97–3 (2) *	97–3 (3) *	96–4 *	95–5 *
Fatty acids	Acid 9:0	0.25 ± 0.02	-	-	-	-	-
	Acid 18:0	0.04 ^a^	-	0.13 ^a^	-	0.01 ^a^	-
	Acid 18:1	0.15 ^a^	-	0.56 ± 0.06	-	-	-
	Acid 18:2	0.51 ± 0.05	-	-	-	-	-
Fatty alcohols	Alcohol 16:0	0.40 ± 0.02	-	-	-	-	-
**Terpenes and Terpenoids**							
	Terpineol	-	-	0.02 ^a^	-	-	-
	*p*-menthane-1,8-diol	0.06 ^a^	-	-	-	-	-
Di-and triglycerides							
	Diglycerides	0.45 ± 0.04	-	0.06 ^a^	-	-	0.01 ^a^
	Triglycerides	0.13 ^a^	-	-	-	-	0.01 ^a^
**Resin Acids**							
Resin acids	Isopimaric acid	0.01^a^	-	-	-	-	-
	Unidentified RT = 22:03-27:80	0.11 ^a^	0.02 ^a^	0.02 ^a^	0.02 ^a^	-	-
Modified resin acids	X-OH-7oxoDHA acid	-	-	0.03 ^a^	0.01 ^a^	-	-
	Dihydroxy-DHA acid	1.39 ± 0.21	-	-	-	-	-
	9-hydroxystearic acid	0.01 ^a^	-	0.01 ^a^	-	-	-
	X-hydroxyabietic acid	0.02 ^a^	-	-	-	-	-
**Steroids**							
	Sitosterol	0.17 ± 0.03	-	0.59 ± 0.09	-	-	-
	Unidentified RT = 28:04-30.69	-	-	0.05 ^a^	-	-	-
**Phenolic Compounds**							
Phenols	Vanillin	-	-	0.07 ^a^	-	0.01 ^a^	-
	Rhododendrol	0.01 ^a^	-	-	-	-	-
	3,4-dihydroxybenzaldehyde	0.01 ^a^	-	-	-	-	-
	3-methoxy-4-OH-cinnamaldehyde	0.06 ^a^	-	0.05 ^a^	-	-	-
	Gallic acid	0.32 ± 0.02	0.01 ^a^	-	-	0.01 ^a^	-
	Protocatechuic acid	0.13 ^a^	-	-	-	-	-
	Tyrosol	0.24 ± 0.01	-	-	-	-	-
	Dihydroconiferyl alcohol	0.28 ± 0.01	-	0.04 ^a^	-	-	-
	Syringic acid	0.05 ^a^	-	-	0.01 ^a^	-	-
	Vanillic acid	0.07 ^a^	-	0.01 ^a^	0.01 ^a^	-	-
	4-hydroxybenzoic acid	0.13 ^a^	-	-	-	-	0.01 ^a^
Phenolic acid	Sinapyl alcohol	0.24 ± 0.02	-	0.04 ^a^	0.01 ^a^	-	-
	1-guaiacyl-3-OH-1-propanone	0.03 ^a^	-	-	-	-	-
	1-guaiacyl-2-OH-1-ethanone	0.03 ^a^	-	0.01 ^a^	-	-	-
	1-syringylglycerol	0.04 ^a^	-	0.01 ^a^	0.03 ^a^	0.01 ^a^	0.01 ^a^
	1-guaiacyl -2,3-diOH-1-propanone	0.04 ^a^	-	0.01 ^a^	-	0.01 ^a^	0.01 ^a^
	1-guaiacylglycerol	0.16 ± 0.01	-	-	-	-	-
	1,3-(bis-guaiacyl)-1,2-propandiol	0.09 ^a^	-	-	-	-	0.01 ^a^
	*P*-hydroxyphenyl glycerol	0.04 ^a^	-	-	-	-	0.01 ^a^
	Coniferyl alcohol	0.25 ± 0.03	-	0.03 ^a^	0.01 ^a^	-	-
Stilbenes and diarylheptanoids	4,4′-diOH-3,3′-dimethoxystilbene	0.01 ^a^	-	-	0.02 ^a^	0.01 ^a^	-
	X-c4h4(oh)3; c5h6(oh)3	0.23 ± 0.02	-	0.02 ^a^	0.01^a^	0.01 ^a^	-
Lignans	Resorcinol	0.10 ± 0.02	-	-	-	-	-
	Olivil	0.01 ^a^	-	0.01 ^a^	-	-	-
	glucopyranoside2[-4(OH)phe]	0.02 ^a^	-	-	-	-	-
	HMR	-	-	-	0.01 ^a^	-	-
	Todolactol	0.17 ± 0.03	-	0.01 ^a^	0.01 ^a^	0.01 ^a^	0.01 ^a^
**Flavonoids**							
tannins	Ellagic acid	0.04 ^a^	-	0.01 ^a-^	0.01 ^a^	-	-
Flavonoids	Pinobanksin	0.01 ^a^	-	-	-	-	-
	Pinocembrin	0.06 ^a^	-	-	0.02 ^a^	0.01 ^a^	-
	(+)-Catechin	0.02 ^a^	-	0.02 ^a^	-	-	0.01 ^a^
	Dihydromyricetin	0.03 ^a^	-	-	-	-	-
	Quercetin	0.05 ^a^	-	0.03 ^a^	-	-	-
	Taxifolin	0.21 ± 0.04	-	-	0.01 ^a^	-	-
	Gallocatechin	0.22 ± 0.02	-	-	0.06 ^a^	0.02 ^a^	-
	3′,4′,5′,3,X-PentaOH-dyhydroflavanol	0.07 ^a^	-	-	-	0.01 ^a^	0.01 ^a^
	Monomethyl gallocatechin	0.02 ^a^	-	-	-	-	-
**Other Compounds**							
Sugar derivatives	Monosaccharides	6.84 ± 1.02	-	0.88 ± 0.08	0.01 ^a^	0.01 ^a^	-
	Sugar acids	1.20 ± 0.09	-	0.06 ^a^	-	-	-
	Sugar alcohol	1.17 ± 0.06	-	0.01 ^a^	-	-	-
Others	Carboxyl acid	1.12 ± 0.14	-	-	-	-	-
	Alpha-lapachone	0.01 ^a^	-	0.02 ^a^	-	-	-
	Dehydro-alpha-lapachone	0.07 ^a^	-	0.10 ± 0.01	-	-	-
	Hydroquinone- β-d-glucopyranoside	0.06 ^a^	-	-	-	-	-
	3-OH-2-methyl-pyran-4-one	0.01 ^a^	-	0.01 ^a^	0.02 ^a^	-	-
	Non-identified	0.13 ± 0.03	-	0.24 ± 0.03	-	-	-

Standard deviation (*n* = 3); ^a^ s.d. < 0.01. * mg of extracts per g of shells.

**Table 5:** Yield of (% of dry mass) total phenolic content and antioxidant activity of the polar extracts and their fractions.

Sample	Fraction	Extraction Yield (%)	Total Phenolic Content (mg GAE/g)	Antioxidant Activity IC50 Values (µg/mL)	ARP (1/IC50)
Hazelnuts shells	**Raw Extract**	-	41.75 ± 0.62 ^a^	7.27 ± 0.19 ^a^	0.14
	97:3 (1)	82.32 ± 4.58 ^a^	31.37 ± 0.48 ^b^	6.34 ± 0.25 ^a^	0.16
	97:3 (2)	5.39 ± 1.35 ^a^	33.62 ± 0.20 ^b^	4.62 ± 0.35 ^a^	0.22
	97:3 (3)	4.21 ± 0.80 ^b^	48.46 ± 0.98 ^a^	2.85 ± 0.58 ^b^	0.35
	96:4	4.21 ± 0.65 ^b^	59.03 ± 1.23 ^a^	2.60 ± 0.32 ^b^	0.38
	95:5	3.88 ± 0.46 ^a^	83.04 ± 3.05 ^a^	1.10 ± 0.07 ^a^	0.91
Walnuts shells	**Raw Extract**	-	13.14 ± 0.18 ^a^	7.82 ± 0.62 ^a^	0.13
	97:3 (1)	0.82 ± 0.07 ^a^	nd	nd	-
	97:3 (2)	85.41 ± 4.65 ^a^	5.1 ± 0.01 ^a^	very low	-
	97:3 (3)	7.57 ± 0.84 ^a^	63.60 ± 1.36 ^a^	7.17 ± 0.44 ^a^	0.14
	96:4	3.51 ± 0.13 ^b^	88.82 ± 4.06 ^a^	3.14 ± 0.22 ^a^	0.32
	95:5	2.70 ± 0.10 ^b^	49.10 ± 0.89 ^a^	5.81 ± 0.26 ^a^	0.17

S.d. (*n* = 3); different letters indicate significant differences at 0.05 level (one way ANOVA): ^a^ Indicates that the difference of the means is significant; ^b^ indicates that the difference of the means is not significant at 0.05 level.
